# The Atrioventricular Conduction Axis in Man and Mouse

**DOI:** 10.3390/jcdd11110340

**Published:** 2024-10-24

**Authors:** Damián Sánchez-Quintana, Yolanda Macías, Jorge Nevado-Medina, Diane E. Spicer, Robert H. Anderson

**Affiliations:** 1 Department of Human Anatomy and Cell Biology, Faculty of Medicine, University of Extremadura, 06006 Badajoz, Spain; yolmacgan@gmail.com (Y.M.); jnevadomedina@gmail.com (J.N.-M.); 2 Heart Institute, Johns Hopkins All Children’s Hospital, St. Petersburg, FL 33701, USA; spicerpath@hotmail.com; 3 Biosciences Division, Newcastle University, Newcastle-upon-Tyne NE2 4HH, UK; sejjran@ucl.ac.uk

**Keywords:** atrioventricular node, bundle of his, bundle branches, fasciculo-ventricular connections, nodoventricular connections, mahaim conduction, superior septal pathways

## Abstract

Those using the mouse for the purposes of electrophysiological research presume that the morphology of the conduction axis is comparable with the human arrangement. As yet, however, to the best of our knowledge, no direct comparison has been made between the species. By comparing our extensive histological findings in the human heart with comparable serially-sectioned datasets prepared from adult murine hearts, we aimed to provide this information. When comparing the gross anatomy, we used three-dimensional datasets of neonatal mice hearts prepared using episcopic microscopy. The overall cardiac architecture is comparable, although the mouse has a persistent left superior caval vein draining via the coronary sinus. An inferior pyramidal space and an infero-septal recess are both present in the murine heart, although they are not as well developed as in the human heart. The overall arrangement of the conduction axis is similarly comparable, albeit with subtle differences reflecting the incomplete wedging of the subaortic outflow tract in the murine heart. Most significantly, the findings in both species reveal the presence of extensive superior septal pathways, which perhaps explain the finding of base-to-apex activation of the ventricular mass known to occur in the murine heart.

## 1. Introduction

The murine heart has been widely used over recent decades as an experimental tool, particularly by those perturbing the genetic pathways to produce congenital cardiac malformations. Others have used genetic manipulation and immunocytochemistry to study the lineages and overall arrangement of the atrioventricular conduction axis [1] and the ramifications of the ventricular conduction system [2]. Those studying the arrangement of the ventricular ramifications of the conduction axis suggest that, although there are discrepancies between the physiology of the conduction systems between the species, the anatomical arrangement is basically the same. Similar conclusions regarding the anatomical arrangements were reached by those who studied the development of the overall axis [1]. We also emphasized the basic similarities between the arrangements as found in man and mouse when comparing the findings with other animal species, such as dogs, pigs, and bovines, in which we pointed to major differences in the course of the ventricular components of the axis relative to the aortic root [3]. In that review, however, we also pointed to the subtle differences to be found between man and mouse, such as the arrangement of the membranous septum. In another of our reviews published at that time, we focused on the potential significance of the superior septal pathways [4], as initially described by Mahaim [5]. We did not discuss the potential presence of such pathways in the murine heart. We are now aware that, should these pathways be present, the mouse heart may well serve as a useful testbed to examine the consequences of so-called selective pacing of the ventricular conduction tissues [6]. It was quite some time ago, in fact, that the group working in Utrecht emphasized that, in the mouse heart, unlike most mammals, ventricular activation occurred in a base-to-apex fashion, rather than the apex-to-base pattern found in mammals with bundle branches insulated from the underlying ventricular septal myocardium [7,8]. In a subsequent investigation of the rat heart, in which the same group identified the insulating tissues that separate the specialized cardiomyocytes of the ventricular conduction system from the adjacent working myocardium, they argued that such insulating sheaths were not present in the murine heart [9]. This finding, however, is in conflict with our own initial examination of histological sections from the murine heart, in which we did find the bundle branches themselves to be insulated from the septal myocardium. The possibility that the septum might be activated via the “paraspecific” system initially described by Mahaim [4], moreover, had not been mentioned by the Utrecht group. This was despite them having cited the investigation of the murine heart by Lev and Thaemert [10], in which the presence of the superior septal pathways had been confirmed. The need to clarify the similarity between the architecture of the atrioventricular conduction axis in man and mouse now becomes more important since other investigators are making in silico comparisons between the species [11]. Such simulations will obviously be more accurate if detailed knowledge can be provided of the specific similarities and differences between the two species. With this in mind, therefore, we have revisited our recent detailed studies of the atrioventricular conduction axis in the human heart [12,13,14], making direct comparisons with new histological datasets prepared from murine hearts. Using three-dimensional datasets available from neonatal murine hearts prepared using episcopic microscopy [15], we have then compared and contrasted the gross cardiac anatomy between the species.

## 2. Materials and Methods

For the purposes of our comparisons, we revisited the numerous datasets at our disposal of serially sectioned human hearts stained using the trichrome technique [12,13,14]. In particular, we re-examined the datasets from the 21 hearts specifically assessed to determine the presence of superior septal pathways. Connections between the conduction axis and the ventricular septal crest were found in 14 of these hearts. We then re-evaluated a human heart from a 43-year-old adult sectioned in its short axis, again with the sections stained with trichrome, along with a similar heart from a fetus of 30 weeks. So as to compare the findings with the arrangement of the conduction axis in the murine heart, we prepared new serially sectioned datasets from 12 adult mice cut in both the short axis of the ventricular mass and in the frontal plane. As with the human datasets, the sections had been stained using the trichrome technique. So as to make comparisons in terms of gross anatomy, we took advantage of the large number of photographs we hold showing the features of the atrial and ventricular chambers, and the atrioventricular junctions, in the human heart. We compared these findings with images obtained from the 6 three-dimensional datasets prepared from neonatal mice by Dr. Timothy Mohun using episcopic microscopy [15]. The datasets were interrogated using Horos software version 4.0.1, permitting us to cut sections directly comparable with the existing photographs of the human hearts.

## 3. Results

### 3.1. The Gross Anatomy of the Human and Murine Atrioventricular Junctions

**Figure 1:**
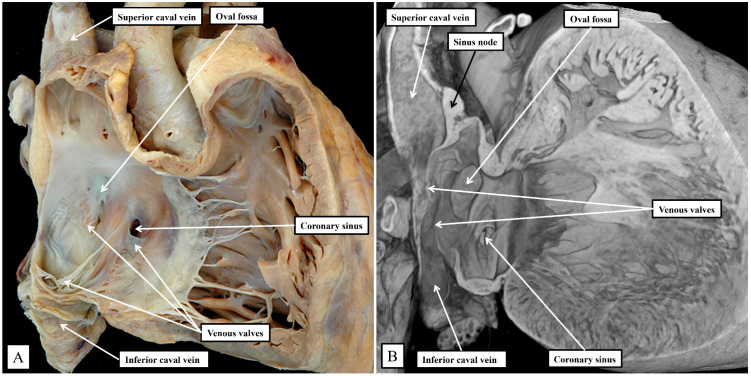
(**A**) shows the features of the morphologically right atrium and ventricle of the human heart, having removed their parietal walls, with the heart shown in an attitudinally appropriate position. (**B**) shows an image from an episcopic dataset from a neonatal mouse heart sectioned to parallel the arrangement as seen in (**A**). The features are basically the same, although, as explained in the text, there are subtle differences that impinge on the arrangement of the atrioventricular conduction axis.

Removing the parietal walls of the right-sided chambers provides an en-face view of the septal atrioventricular junctions, and the septal surfaces of the morphologically right atrium and right ventricle (Figure 1A). A view of an episcopic dataset from a neonatal mouse prepared in a comparable fashion shows the similarities when the murine heart is positioned so as to parallel the human heart as viewed in an attitudinally appropriate fashion (Figure 1B).

**Figure 2:**
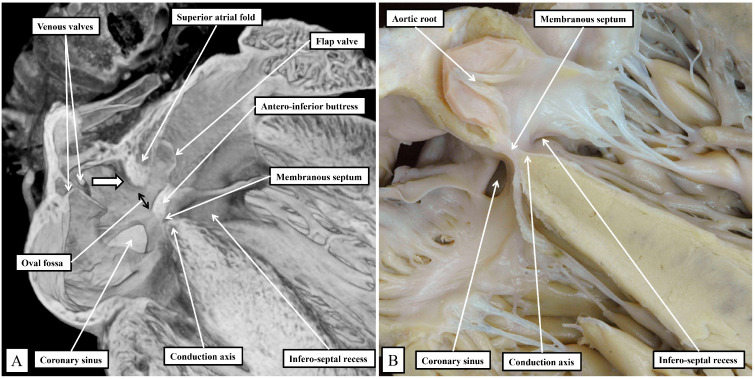
Sections taken across the membranous septum show the markedly different relationships between this part of the septum and the oval fossa on the murine heart (**A**) when compared to the human heart (**B**). The section of the murine heart also shows how the right and left venous valves create a funnel (white arrow with black borders) that directs the flow from the inferior caval vein, the orifice of the vein is not seen in the section, towards the oval fossa. Note the extensive flap valve of the fossa impinging on the roof of the left atrium. The plane of sectioning does not show the fold found inferior in the murine heart as one of the rims of the oval fossa. From the stance of our current study, in both sections, it is possible to visualize the atrioventricular conduction axis sandwiched between the membranous septum and the crest of the muscular ventricular septum.

As is shown in the photographs, when viewed attitudinally, the features are comparable. There are, however, subtle differences which impact on the location of the components of the atrioventricular conduction axis. Perhaps the most significant difference is the presence of a persistent left superior caval vein in the murine heart. This channel opens inferiorly into the vestibular region of the right atrium through an enlarged orifice of the coronary sinus (Figure 1B). In the human heart, the coronary sinus opens much more superiorly within the cavity of the right atrium (Figure 1A). The superior and inferior caval veins enter the systemic venous sinus in a comparable fashion in both species, but the opening of the venous sinus to the morphological right atrium is more dorsal in the murine heart (Figure 1B). The junction between the venous sinus and the remainder of the atrium is then much more obvious in the murine heart since the venous valves are much better preserved, with obvious right and left venous valves coming together at a superior commissure attached within the atrial appendage by the prominent pectinate muscle known as the septum spurium (Figure 1B). In the human heart, it is usually only parts of the right venous valve that persist. These remnants are the Eustachian valve, which partially guards the entrance of the inferior caval vein, and the Thebesian valve, which guards the opening of the coronary sinus (Figure 1A). The two valves come together at the area of the atrial wall separating the orifice of the coronary sinus from the oval fossa. Usually described as the sinus septum, the area is a fold between the walls of the inferior caval vein and the coronary sinus. The area of union between the valves then continues as a tendinous structure that runs within the antero-inferior buttress of the oval fossa. As we will describe, this fibrous entity, known as the tendon of Todaro, forms a boundary of the triangle of Koch, itself providing a landmark to the site of the atrial components of the conduction axis. The situation is more obvious in the murine heart since venous valves marking both the right and left borders of the systemic venous sinus remain subsequent to birth (Figure 1B). In the human heart, nonetheless, with careful examination, it is sometimes possible to recognize small remnants of the left venous valve plastered onto the septal surface of the oval fossa and its surrounds. In the murine heart, the right and left venous valves form a tunnel that directs the flow from the inferior caval vein toward the oval fossa. The superior extension of both valves into the area between the coronary sinus and the oval fossa is also much more marked, with a triangular area such as described initially by Koch also to be found in the mouse heart (see below). There are also subtle differences in the arrangement of the oval fossa between the species. In the human heart, the superior rim of the fossa is an obvious infolding between the attachments of the superior caval vein to the right atrium, and the right pulmonary veins to the left atrium. This arrangement is not found in the murine heart since the pulmonary veins open to the morphologically left atrium through a common orifice located inferiorly rather than superiorly, with a fold present in the dorsal wall, rather than superiorly as found in the human heart. The flap valve of the oval fossa in the murine heart closes against the roof of the atrium, such that the dorsal fold is not obvious (Figure 2A), rather than directly against the superior interatrial fold as is the case in the human heart.

The superior fold is often incorrectly identified as the “septum secundum”. In both species, however, the true second atrial septum can be identified as the antero-inferior buttress of the oval fossa, well seen in the murine heart when frontal sections are taken through the fossa (Figure 2A). Figure 2A then serves to emphasize another major, but subtle, difference between the species that achieves significance when considering the location of the conduction axis. This is the presence of a much-reduced membranous septum in the murine heart, with the septum itself located inferior to the antero-inferior buttress (Figure 2A). A comparable section through the membranous septum in the human heart cuts through the aortic root, rather than the oval fossa. The fossa in the human heart is located inferiorly and posteriorly relative to the membranous septum and is not seen in cuts made in the frontal plane (Figure 2B). The difference in the location of the membranous septum between the species can be related, in part, to the morphology of the coronary sinus.

**Figure 3:**
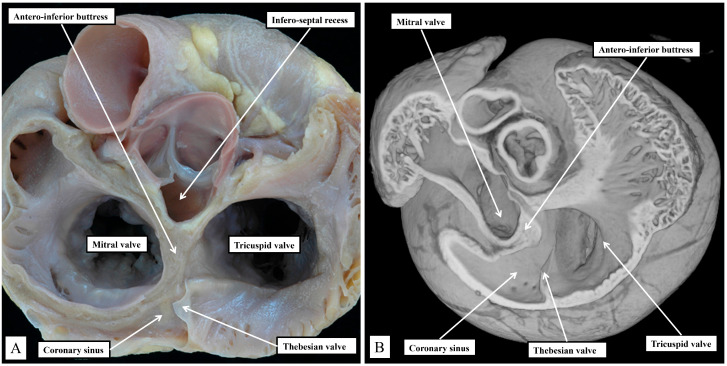
Panel (**A**) Human heart, Panel (**B**) Murine heart. The images are prepared by removing the atrial walls and the arterial trunks and photographing the atrioventricular junctions from the atrial aspect. The marked differences are discussed in the text.

In the human heart, the coronary sinus serves only as the conduit for the return of the greater part of the venous drainage of the heart since the left superior caval vein has usually regressed in the human heart, with its walls persisting only as the ligament of Marshall. In the murine heart, as already explained, it is the norm for the left superior caval vein to persist, and to open to the right atrium through the orifice of the coronary sinus. This produces a marked difference in the relationship of the orifices of the right and left atrioventricular junctions, guarded by the tricuspid and mitral valves (Figure 3).

**Figure 4:**
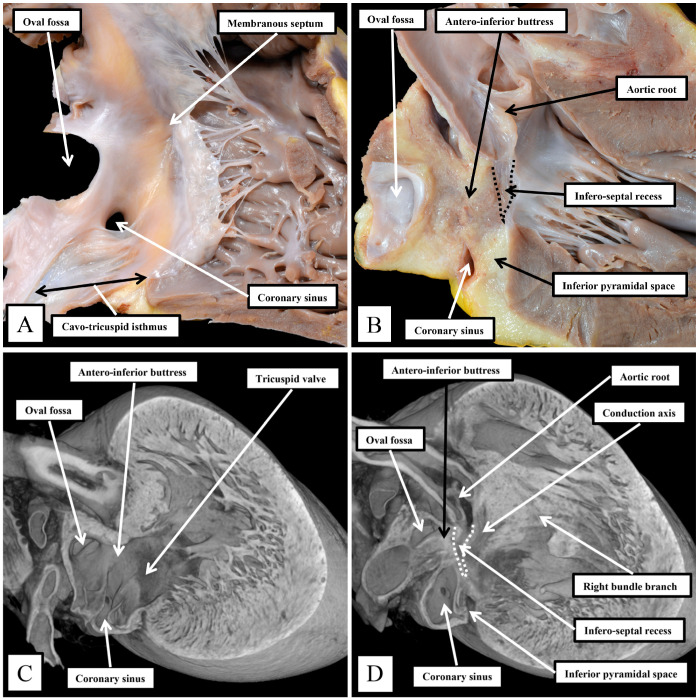
The images show the interrelationships of the apexes of the inferior pyramidal space, extending superiorly, and the infero-septal recess, extending inferiorly. It is the adjacency of the two spaces that makes it possible for the atrioventricular conduction axis to penetrate from the atrioventricular node through the plane of insulating fibrous tissue to become the non-branching atrioventricular bundle. The arrangement in the human heart is revealed by dissecting away the septal surface of the right atrium and the underlying ventricular septum. (**A**) shows the right-sided view of the removed components. (**B**) then shows how the cut extends across the rims of the oval fossa and into the inferior pyramidal space but also into the cavity of the left ventricle, thus showing its parietal components. The situation has been replicated in the murine heart by cutting an episcopic dataset, as shown in (**C**,**D**). The cut in (**D**), however, passes through the crest of the muscular ventricular septum.

In the murine heart, furthermore, as previously noted by Icardo and colleagues [16], it is often difficult to find a third leaflet guarding the right atrioventricular junction (Figure 3B), whereas, in the human heart, the right atrioventricular valve usually closes in trifoliate fashion, even though it may be difficult to find a zone of apposition supported by discrete papillary muscles between the inferior and the antero-superior leaflets. The other major difference to be noted in the junctional arrangements, however, is the much deeper “wedging” of the aortic root between the leaflets of the mitral valve and the septum (Figure 3A). The combination of the enlarged orifice of the membranous septum and the lack of aortic wedging in the murine heart means that, although there is an infero-septal recess between the aortic leaflet of the mitral valve and the septum, it does not extend beneath the antero-inferior buttress of the atrial septum to the same extent as is seen in the human heart [17]. The inferior pyramidal space, furthermore, does not extend as far superiorly in the murine heart as in the human heart. This, again, reflects the differing relationships between the orifices of the right and left atrioventricular junctions (Figure 3), and the presence in the murine heart of the persistent left superior caval vein (Figure 3B). In the human heart, the inferior pyramidal space extends beneath the antero-inferior buttress of the atrial septum, being confined by the diverging walls of the right and left atrial vestibules [17]. In the murine heart, the vestibule of the right atrioventricular junction itself extends much more inferiorly, having to receive the enlarged orifice of the coronary sinus (Figure 3B). In the human heart, it is the extensive superior extent of the inferior pyramidal space that serves to place the septal wall of the right atrial vestibule adjacent to the inferior extent of the infero-septal recess of the left ventricular outflow tract [17]. These relationships, as we will explain, then determine the equally subtle differences in the location of the atrioventricular conduction axis relative to the septal landmarks in the human heart as opposed to the murine heart. The differences can be demonstrated by making a cut that removes the right atrial walls of the human heart, leaving behind the rims and floor of the oval fossa, but extending through the antero-inferior buttress of the atrial septum so as to cut into the infero-septal recess (Figure 4A). The part of the heart thus revealed, having removed the ventricular septal components to show the parietal components of the left ventricle, then shows how the apex of the inferior pyramidal space, extending superiorly, meets the inferiorly directed apex of the infero-septal recess (Figure 4B).

**Figure 5:**
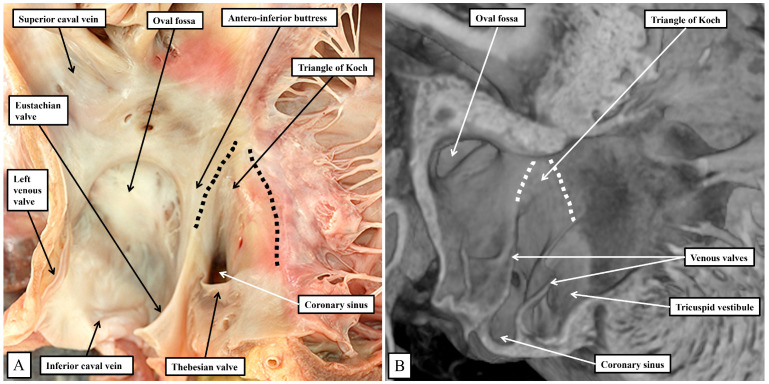
The images show the septal surface of the right atrioventricular junction in the human heart (**A**) compared to the murine heart (**B**). The dashed lines in panels A and B delimit the Koch triangle. In both species, the extension of the fold of the venous valve between the orifice of the coronary sinus and the oval fossa produces the border of the triangle named for Koch. As we will describe, in both species, the atrioventricular node lies beneath the septal vestibule at the apex of the triangle formed with the hinge of the septal leaflet of the tricuspid valve.

It is the septal surfaces of the right atrium, however, that provide the key landmarks to the location of the atrial components of the atrioventricular conduction axis. And here again, the arrangements between the species are similar, albeit with subtle differences. These differences again reflect, for the larger part, the presence in the murine heart, of the persistent left superior caval vein, and the enlarged orifice of the coronary sinus. This means, as already discussed, that the orifice of the coronary sinus, guarded by the Thebesian valve, is positioned more superiorly in the human heart when compared with the arrangement seen in the murine heart. In both species, nonetheless, it remains possible to recognize how the Eustachian and Thebesian valves come together, extending to run as the tendon of Todaro within the atrial walls interposing between the orifice of the coronary sinus and the oval fossa. The arrangement is more obvious in the murine heart since the orifice of the coronary sinus itself extends to occupy the inferior component of the chamber when viewed in an attitudinally appropriate arrangement for the human heart (Figure 5).

In both species, therefore, it is possible to recognize the important triangle as initially described by Koch [18]. The apex of the triangle, in the human heart, is adjacent to the infero-septal recess of the left ventricular outflow tract, albeit the spaces are separated by the atrioventricular septal structures (Figure 4B). In the murine heart, in contrast, the aortic root is located more superiorly when viewed in an attitudinally appropriate fashion, reflecting the lack of wedging when compared to the human heart (Figure 3). The greater inferior extent of the orifice of the coronary sinus in the murine heart (Figure 5B) then produces a major difference in the structure of the cavo-tricuspid isthmus. In the human heart, the isthmus incorporates an extensive sub-Thebesian sinus (Figure 5A). In the murine heart, again when considered attitudinally, the vestibular sinus is anterior, rather than inferior, when assessed relative to the Thebesian valve (Figure 5B). All of these subtle changes between the human and murine hearts in terms of the gross anatomy are then significant when set against the location of the atrioventricular conduction axis in the two species.

### 3.2. The Atrioventricular Conduction Axis of the Human and Murine Hearts

**Figure 6:**
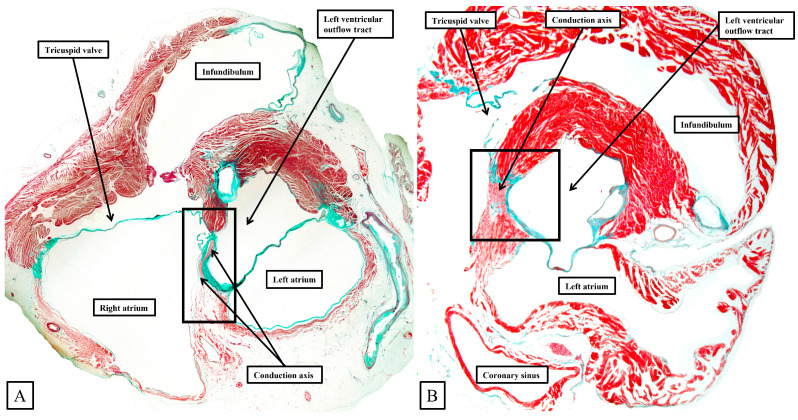
Histological sections have been taken across the short axis of the atrioventricular junctions of the human heart, as seen in (**A**), and compared with the arrangement in the murine heart, as shown in (**B**). The areas as shown within the black rectangle in (**A**), and the black square in (**B**), are then shown in a series of serial sections in Figure 7 and Figure 8 for the human heart and Figure 9 for the murine heart.

The significance of the deeply wedged location of the aortic root, along with the extensive superior extent of the inferior pyramidal space, when compared to the arrangement as found in the murine heart, are well appreciated when examining short axis sections of the atrioventricular junctions in the two species. In both species, the penetration of the conduction axis from the atrial to the ventricular segments is found towards the center of the short axis. As we have already discussed, in the human heart, the extensive inferior pyramidal space is bordered by the diverging walls of the right and left atrial vestibules. This is well seen in the short axis section shown in Figure 6A. In the murine heart, in contrast, as shown in Figure 6B, the antero-inferior buttress of the atrial septum is more directly apposed to the crest of the muscular ventricular septum. As we showed in Figure 5, although an infero-septal recess is present in both species, and its inferior apex extends to reach the superiorly extending apex of the inferior pyramidal space, the overlap between the two spaces is much greater in the human heart than in the murine heart (Figure 6).

As can readily be seen in Figure 6B, the vestibular inputs in the murine heart enter the compact atrioventricular node directly from the antero-inferior buttress of the atrial septum, reflecting the much smaller inferior pyramidal space of the mouse. As we will also see, nonetheless, in both species the transition from the atrial to the ventricular segments occurs as the conduction axis passes beneath the area of fibrous continuity between the leaflets of the mitral and tricuspid valves, albeit with this area reinforced in the murine heart by a contribution of fibrous tissue from the analog of the tendon of Todaro.

### 3.3. The Specifics of the Atrioventricular Conduction Axis of the Human Heart

**Figure 7:**
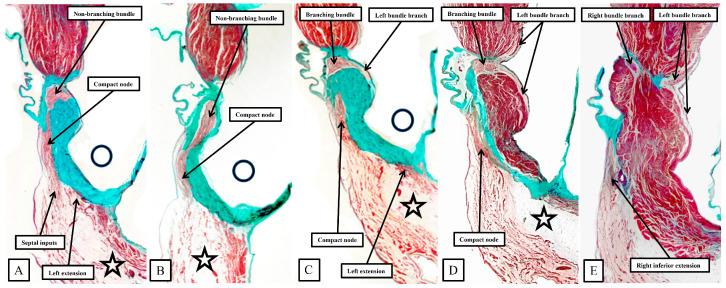
The serial sections from the human heart, representing sequential sections along the rectangle shown in Figure 6A, reveal the components of the atrioventricular conduction axis. (**A**) is the most superior section when considered relative to the base and apex of the ventricular cone, but the lower part of the section shows the inferior part of the short axis itself. (**B**–**E**) is that as explained in the text of Figure 7 they are sequential histological sections. The star shows the inferior pyramidal space, with the open circle showing the infero-septal recess. See text for discussion.

It was the stellar investigation of Tawara that clarified the very existence of the atrioventricular conduction axis [19]. In his monumental monograph, Tawara described the findings in several animal species, albeit not paying any attention to the murine heart. Our own findings in the human heart [14] are very much an endorsement of his observations. As can be inferred from the illustrations provided by Tawara, when the triangle of Koch is considered in an attitudinally appropriate fashion, and when serial sections are assessed moving inferiorly to superior across the inferior pyramidal space, it is possible to recognize how the atrial working myocardium of the atrial vestibules give rises to the right and left inferior extensions of the atrioventricular node, with the rightward extension extending significantly inferiorly to pass within the septal isthmus between the orifice of the coronary sinus and the hinge of the septal leaflet of the tricuspid valve [13]. It is these inferior extensions that constitute the slow pathways into the node [20]. In Figure 7, we are showing comparable serial sections, taken in a short axis from the same dataset as shown in Figure 6, but placed so as to run superiorly from the base of the ventricular cone (Figure 7A) towards the ventricular apex (Figure 7E). The right inferior nodal extensions are thus seen in the deepest section (Figure 7E).

**Figure 8:**
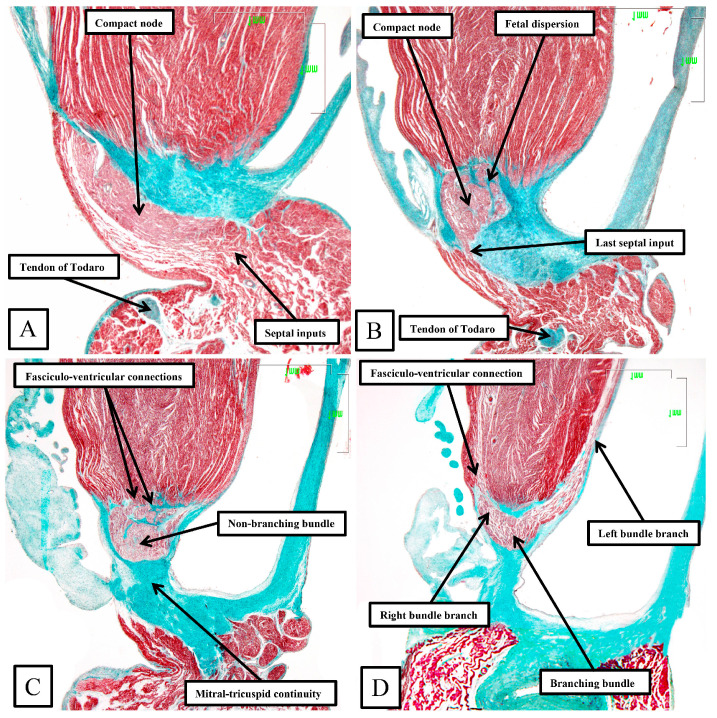
The panels (**A**–**D**) are sequential histological sections show the transition from the atrioventricular node to the ventricular components of the atrioventricular conduction axis as seen in a prematurely born human infant. The serial sections, although cut in the frontal plane, have been orientated to match the arrangement shown in Figure 7. See text for further discussion.

Taken together, the sections show how the inferior extensions of the node, merging with the working myocardium of the atrial vestibules, come together to form the compact atrioventricular node. In the human heart, the compact node is set as a half-oval against the prominent fibrous tissues of the atrioventricular junction (Figure 7A,B). The compact node then receives direct inputs from the antero-inferior buttress of the atrial septum (Figure 7C,D). It is the last input from the atrial septum that forms the fast pathway into the node [20]. The transition from the atrial to the ventricular components of the conduction axis, along with the arrangement of the ventricular components, is better seen when sections are cut in the frontal plane (Figure 8).

The frontal sections show well how the compact node is formed as a half-oval of histologically specialized cells supported on the so-called central fibrous body. The fibrous tissue is derived from the atrioventricular cushions that, in the developing heart, divide the atrioventricular canal into its right and left parts. As was described initially by Keith and Flack [21], the insulation of the axis from the atrial myocardium is provided by the union of the fibrous tissues of the cushions that develop into the leaflets of the tricuspid and mitral valves. This is well seen in Figure 8B,C, with the atrial myocardium still joining the node in Figure 8B, but insulated from the axis in Figure 8C. It was this feature of insulation of the axis from the atrial myocardium that Tawara [18] used as the criterion for distinction between the atrioventricular node (Figure 8B) and the non-branching atrioventricular bundle (Figure 8C). As can also be seen in Figure 8C, although the axis is insulated from the atrial myocardium, connections still exist between the non-branching bundle and the crest of the muscular ventricular septum. These are the fasciculo-ventricular connections, or superior septal pathways, that were identified by Mahaim as the paraspecific system for atrioventricular conduction [4]. In the human heart, having taken a short course along the crest of the muscular ventricular septum, the axis then branches to give rise to the right and left bundles (Figure 8D). Additional fasciculo-ventricular connections were observed in two-thirds of our datasets [5], as seen also in Figure 8D, between the rightward margin of the branching bundle and the crest of the muscular ventricular septum.

### 3.4. The Specifics of the Atrioventricular Conduction Axis of the Murine Heart

**Figure 9:**
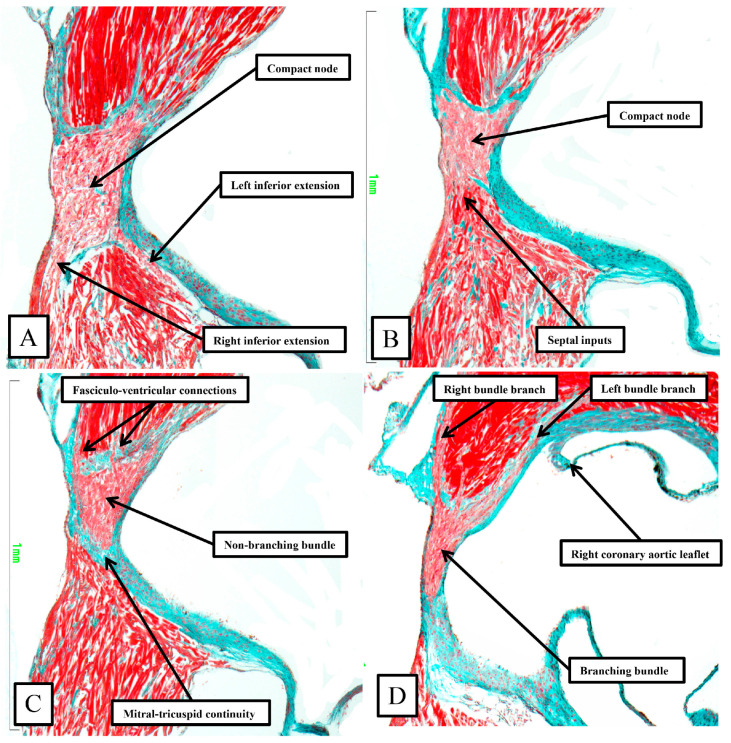
Panels (**A**–**D**) are sequential histological sections show the transition from the atrioventricular node to the ventricular components**.** The images, taken from the murine heart sectioned in the short axis of the ventricular cone (see square in Figure 6), show the basic arrangement of the atrioventricular conduction axis in the murine heart.

Although having the same basic arrangement as shown in Figure 7 and Figure 8 for the human heart, there are important differences in the murine heart (Figure 9).

**Figure 10:**
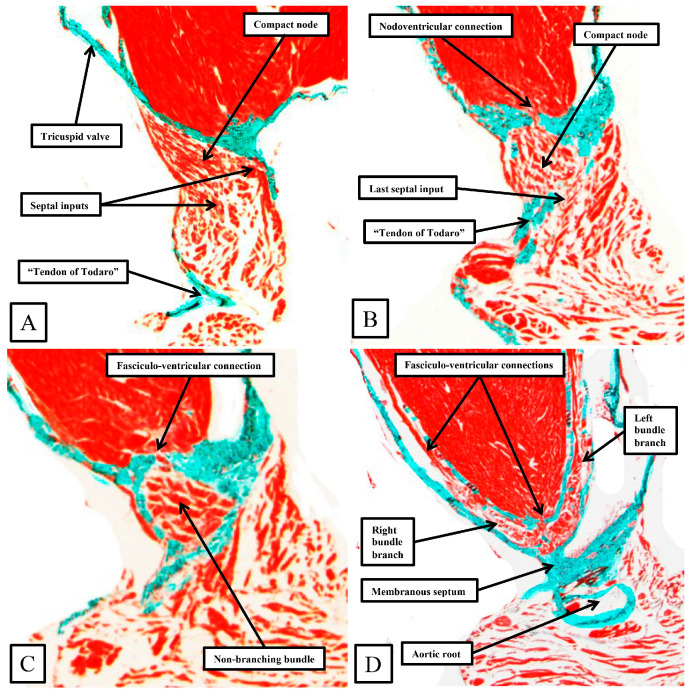
Panels (**A**–**D**) are sequential histological sections show the transition from the atrioventricular node to the ventricular components. This series of sections from a murine heart, stained with the trichrome technique, shows the arrangement of the conduction axis when the heart is cut in the frontal plane. The images are orientated, however, to match the arrangements shown in Figure 6, Figure 7, Figure 8 and Figure 9. See text for further discussion.

As already shown in Figure 5 and Figure 6, the apex of the inferior pyramidal space in the murine heart does not overlap the infero-septal recess of the left ventricular outflow tract to the same extent in the murine as in the human heart. Because of this, there is less separation of the inputs to the compact atrioventricular node from the right and left atrial vestibules. The histologically specialized extensions, nonetheless, can still be distinguished from the working atrial myocardium on the basis of the staining characteristics (Figure 9A). The compact node is more square in the murine heart, receiving extensive direct inputs from the antero-inferior buttress of the atrial septum (Figure 9B). As in the human heart, nonetheless, the axis becomes insulated from the atrial myocardium as it passes beneath an area of fibrous continuity between the leaflets of the mitral and tricuspid valves (Figure 9C). And, again, as in the human heart, extensive fasciculo-ventricular connections, or superior septal pathways, can be identified between the non-branching bundle and the crest of the muscular ventricular septum (Figure 9C). In the murine datasets, furthermore, the connections were observed in all the datasets examined thus far. Following the arrangement in the human heart, albeit with a longer non-branching component, the axis then branches on the crest of the muscular septum. The similarities with the human heart are further seen in that the right bundle branch runs as a narrow cord as it descends the muscular ventricular septum, whereas the left bundle branch takes a broader origin from the axis, ramifying in trifascicular fashion as its branches move towards the ventricular apex. As can be seen in Figure 9D, however, the branches themselves, as in the human heart, are enclosed in fibrous sheaths in the murine heart as they descend the ventricular septum. There are, nonetheless, additional direct connections between the rightward margin of the branching bundle and the septal crest before the right bundle itself becomes fully insulated. These connections produce additional superior septal pathways. These relationships can then be confirmed when murine hearts are cut in the frontal plane (Figure 10).

When compared to the arrangement in the human heart, the antero-inferior buttress of the atrial septum can be seen to lie much more in an edge-to-edge fashion relative to the crest of the muscular septum, with a better-formed central fibrous body in the human heart (compare Figure 8A and Figure 10A). The last connection between the atrial myocardium and the axis, however, is still formed just prior to the insulation of the axis by the fibrous continuity formed between the leaflets of the tricuspid and mitral valves, albeit with this insulation reinforced in the murine heart by a contribution from the tendon of Todaro (Figure 10B,C). Connections are present in the murine heart between both the compact node (Figure 10B) and the non-branching bundle (Figure 10C) and the crest of the muscular ventricular septum. The non-branching bundle itself is then much longer in the murine heart, reflecting the greater distance between the poorly formed membranous septum and the aortic root. The axis does then branch on the crest of the septum, with the right and left fascicles well-insulated from the underlying septal myocardium (Figure 10D). Additional direct connections can be identified, nonetheless, between the origin of the insulated cord-like right bundle branch and the working septal cardiomyocytes (Figure 10D).

### 3.5. The Potential Significance of the Superior Septal Pathways

**Figure 11:**
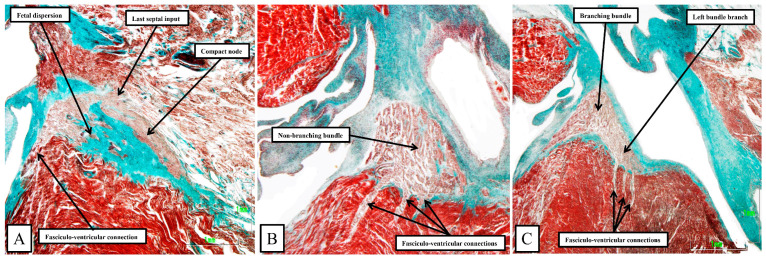
The images are taken from serial sections of a human fetus of 30 weeks gestation. The heart was sectioned in the sagittal plane, and the images are orientated with the atrial septum to the top. The sternocostal surface is seen to the left hand, with the diaphragmatic surface to the left hand. (**A**) shows a long axis section of the compact node and its transition to the penetrating bundle, with the last septal connection also seen. There is fetal dispersion of the nodal cardiomyocytes, along with a direct fasciculo-ventricular connection. (**B**,**C**) show extensive fasciculo-ventricular pathways, distinguished histologically from the septal myocardium, which pass through gaps in the insulating tissues to produce multiple superior septal pathways, as initially described by Mahaim [4].

During early cardiac development, the ventricular walls are made up, in their larger part, of a meshwork of trabecular myocardium [22]. At this initial stage, there is only a very thin compact component of the parietal ventricular walls. The septum, however, which forms concomitant with the “ballooning” of the ventricular apical components, is already forming by coalescence of the pre-existing trabeculations. With ongoing development, the surface layers of the septum specialize to form the ventricular bundle branches, with the trabeculations also coalescing to produce the papillary muscles of the atrioventricular valves and the prominent septomarginal trabeculation of the right ventricle [23]. The apical trabeculations also persist in the human heart, developing in such a way as to permit distinction of the morphologically right and morphologically left chambers, although the difference in trabecular pattern is less well marked in the murine heart. The developing bundle branches themselves take their origin from a further specialized component that can be recognized as a ring surrounding the primary interventricular foramen [23]. When first formed, there are extensive myocardial connections between the ring and the crest of the developing muscular ventricular septum. It is the development of the fibrous tissues that disrupts most, but not all, of these pathways. The ring is also continuous, initially, with the rightward margin of the atrioventricular canal myocardium. With the expansion of the atrioventricular canal and formation of the secondary interventricular foramen, the ventricular component of the ring becomes continuous with the atrioventricular canal myocardium at the crux of the heart, with the transition sandwiched between the atrioventricular cushions luminally and the tissues of the inferior atrioventricular groove epicardially. It is the transition from beneath the cushions, which become the leaflets of the atrioventricular valves and the central fibrous body, and the tissues of the atrioventricular groove, which serves to provide the insulation of the atrioventricular node as it becomes the non-branching atrioventricular bundle. So as to function as a specialized conducting system, however, it is also necessary for the developing pathways to become insulated from the crest of the ventricular septum. Such insulation is not fully developed at the end of the embryonic period of development. Thus, towards the end of the embryonic period, extensive connections remain between both the developing compact atrioventricular node and the non-branching bundle and the crest of the muscular ventricular septum. Throughout the earliest part of the fetal development, such connections can still be identified between the conduction axis and the crest of the muscular ventricular septum. These pathways initially form extensive nodoventricular and fasciculo-ventricular pathways. Towards the end of the fetal period, the nodoventricular pathways become less evident, being dispersed within the developing fibrous tissues of the central fibrous body, although discrete pathways can still be identified towards the end of the fetal period [24]. The pathways between the non-branching bundle and the crest of the muscular ventricular septum, however, continue to remain visible as prominent structures (Figure 11).

## 4. Discussion

Our retrospective analysis of our extensive studies of the arrangement of the atrioventricular conduction axis of the fetal and postnatal human heart show marked similarities, but subtle differences, with the pattern to be found in the murine heart. This is in keeping with the conclusions reached by those who studied the development of both the atrioventricular conduction axis itself [1] and also the ramifications of the ventricular conduction system [2]. The current analysis extends our own comparative study of the conduction axis in various species. We had commented previously on the similarity between the arrangements found in man and mouse, contrasting this with the major differences to be found in canine, porcine, and bovine species [3]. In our initial report, however, we had paid scant attention to the superior septal pathways. It is of note that Lev and Thaemert had already provided an overall account of the location of the conduction system of the murine heart [10]. Our current findings are in keeping with their descriptions [4]. Mahaim suggested that the pathways could function as a “paraspecific” system for atrioventricular conduction [4]. In the book in which he first described the pathways, published in the French language [25], he provided electrocardiographic evidence to support his concept. Perhaps surprisingly, the superior septal pathways have subsequently received minimal attention in terms of their potential role as a paraspecific system for conduction, albeit both the nodoventricular and fasciculo-ventricular pathways are well-recognized as rare substrates for ventricular pre-excitation [26]. Even with regard to pre-excitation, however, the name of Mahaim is more usually associated with the so-called atrio-fascicular tracts, although Mahaim himself never described these pathways [27]. The pathways have now received new attention with the realization that they may, indeed, function as a paraspecific system for ventricular activation during the so-called selective pacing of the ventricular conduction axis. It has been argued that the strength of the pacing stimulus is sufficient to activate the septal pathways, which remain dormant during normal conduction [6]. Our finding that such pathways are ubiquitous in the murine heart, therefore, means that the mouse can now serve as a testbed to establish whether the pathways can, indeed, serve to activate the ventricular mass. There is already potential evidence to indicate that this may be the case. Thus, the group working in Utrecht, in the first decade of the 21st century, had noted that the ventricular mass in the murine heart was activated from base to apex, rather than from the apex, as is the case in all other mammalian species [7,8]. They argued that this phenomenon might reflect the fact that the proximal bundle branches in the murine heart were not themselves insulated from the underlying ventricular septal myocardium, a feature they had demonstrated in the hearts of rats [9]. Our current histological sections stained using the trichrome technique, however, show that the bundle branches in the murine heart, as they descend the ventricular septum, are insulated by fibrous sheaths as they are in the human heart (compare Figure 8 and Figure 10). If the superior septal pathways are, indeed, functioning as a paraspecific system for ventricular activation, as suggested by Mahaim [4], with his findings further endorsed by pacing studies [6], then as suggested above, the mouse heart can serve as a testbed for further electrophysiological studies.

It is the presence of the superior septal pathways in both the human and murine hearts that has been the major focus of our review. It is noteworthy, however, that the overall arrangement of the conduction axis is remarkably similar in the murine and human hearts, almost certainly reflecting the fact that both hearts show an inferior pyramidal space within the atrioventricular junctions, which overlaps the infero-septal recess of the left ventricular outflow tract. These components of the heart have themselves not received the attention they perhaps deserve. It is the differences in the extent of the two components that underscore the subtle differences to be found between the species. In the human heart, furthermore, the extent of both areas can now be established using computed tomography. This will hopefully permit further inferences to be made regarding the potential influence of features such as the rotation of the aortic root within the base of the ventricular mass on iatrogenic damage subsequent to transcatheter replacement of the aortic valve [28].

## Data Availability

The histological datasets of the human and murine hearts were prepared by Professor Sánchez-Quintana and Yolanda Macías, and they are able to make PowerPoints Microsoft Office 2021, available to interested parties by direct communication. Some of the episcopic datasets of murine hearts prepared by Dr. Mohun can now be accessed via the website of the Human Developmental Biology Resource.
